# Phagosomal transport depends strongly on phagosome size

**DOI:** 10.1038/s41598-017-17183-7

**Published:** 2017-12-06

**Authors:** S. Keller, K. Berghoff, H. Kress

**Affiliations:** Department of Physics, University of Bayreuth, Universitaetsstrasse 30, D-95440 Bayreuth, Germany

## Abstract

Macrophages internalize pathogens for intracellular degradation. An important part of this process is the phagosomal transport from the cell periphery to the perinuclear region. Biochemical factors are known to influence the fate of phagosomes. Here, we show that the size of phagosomes also has a strong influence on their transport. We found that large phagosomes are transported persistently to the nucleus, whereas small phagosomes show strong bidirectional transport. We show that dynein motors play a larger role in the transport of large phagosomes, whereas actin filament-based motility plays a larger role in the transport of small phagosomes. Furthermore, we investigated the spatial distribution of dyneins and microtubules around phagosomes and hypothesize that dynein and microtubule density differences between the nucleus-facing side of phagosomes and the opposite side could explain part of the observed transport characteristics. Our findings suggest that a size-dependent cellular sorting mechanism might exist that supports macrophages in their immunological roles.

## Introduction

The internalization of pathogens by macrophage phagocytosis and the subsequent digestion of pathogens within the macrophages are two of the key processes of the mammalian immune system^1^. During the macrophage immune response, cells can differentiate between pathogens, including bacteria or endogenous contents such as apoptotic cells, and decide if an inflammatory or a tolerant response is necessary. Therefore, phagocytosis is an important process for ensuring immune defense and therefore mammalian health^2,3^. Many bacteria can interrupt the phagocytic maturation process to prevent their digestion and can even replicate within the macrophages themselves^2–5^. Common examples for such bacteria include *Mycobacterium* spp.*, Salmonella* spp., *Cryptococcus neoformans, Chlamydia* spp. or *Brucella abortus*; the first two types of bacteria can arrest phagosome maturation and the latter two examples can convert a phagosome into a non-phagosomal organelle^2,6,7^. *Cryptococcus neoformans* manages to escape the phagosome and can extrude itself from macrophages by an unknown mechanism without killing the host^8,9^.

The abovementioned examples show that, in a large number of cases, deviations from normal phagosomal maturation can occur. The range of parameters that can theoretically form the basis for these variations is very large, and biochemical modulations, such as the bacterial expression of effector proteins and lipids, have been shown to play a role^5^. However, the influence of a fundamental and simple parameter that describes one aspect of bacterial variation has barely been investigated to date: the size of the phagocytic target. In addition to its fundamental and simple character, this parameter has the additional advantage of being able to be varied in experiments with a high degree of control in a continuous fashion. Since the transport of a phagosome to the perinuclear region is an important part of the maturation process^10–13^, we investigate in this work the influence of phagosome size on the phagosomal transport behavior.

**Figure 1:**
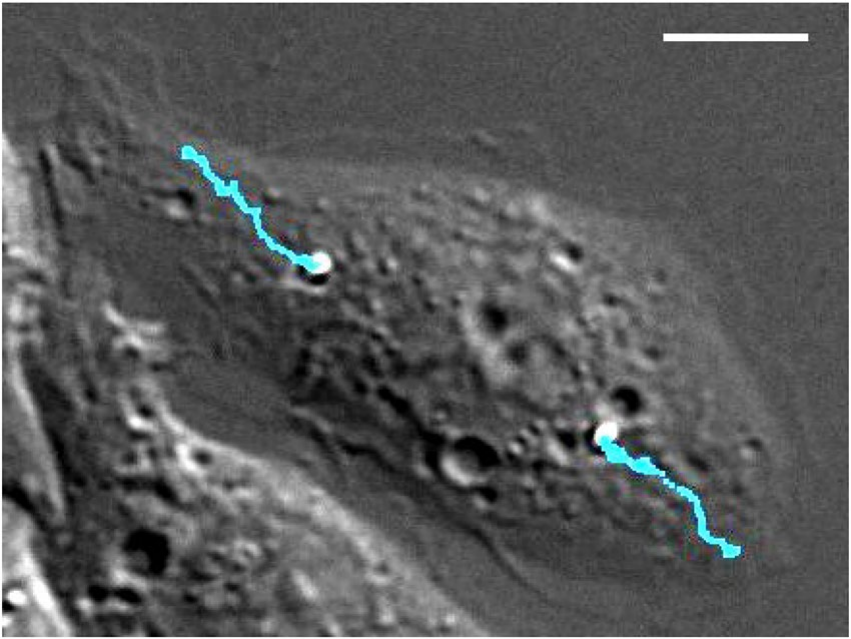
Transport of two phagosomes within a macrophage. Shown is one frame of a DIC time lapse image series of a J774 cell with two phagocytosed IgG-opsonized particles with a diameter of 2 µm. Overlaid in cyan are the trajectories of the two phagosomes, from the beginning of the time series to the shown frame. The trajectory represents approximately 8 min of phagosomal transport. The scale bar is 10 µm.

To date, several studies have investigated the influence of the phagocytic target size on the engulfment process in macrophages^14–17^, but its influence on subsequent phagosome transport is still largely unknown, aside from the finding that phagosome size has an influence on lysosome delivery^14^. Furthermore, it has been found that fibroblasts induce a size-dependent segregation of internalized microspheres^18^. The size of the bacteria that are mentioned as examples above differ, from approximately 1–2 µm in the case of *Brucella abortus* ^19^, to more than 4 µm in the case of *Cryptococcus neoformans* ^20^. For our study, we stayed in this range of naturally occurring phagocytic target sizes and used microparticles with diameters from 1 µm up to 3 µm. The microparticles were opsonized with immunoglobulin G (IgG), which is an established approach to investigate Fcγ-receptor mediated phagocytosis^21,22^. By tracking the particles after internalization into J774 macrophages^23^, we were able to characterize the transport of individual phagosomes in detail (Fig. 1).

We found that phagosomal transport behavior depends strongly on the phagosome size. While large, 3 µm-sized phagosomes showed a very effective, persistent centripetal transport (towards the nucleus), small phagosomes with a diameter of 1 µm, however, showed highly irregular motion patterns, with frequent phases of centrifugal motion (towards the cell periphery). Surprisingly, the large majority of these small phagosomes did not remain in the perinuclear region, which is generally considered the endpoint of the phagosomal transport^10^. Direction changes during transport can be associated with specific cellular functions, such as the removal of phagocytic remnants for antigen presentation at the end of a successful phagosome maturation^11,24–26^.

To investigate the molecular basis for this size-dependent transport behavior, we studied the influence of actin- and microtubule-based motility on phagosomal transport. Whereas microtubules in combination with dynein and kinesin motors are strongly involved in long-range phagosomal transport, actin-based motility has been suggested to be important for short-range movements^3,11,27–29^. We found that actin filament-based motility plays a larger role in the transport of small phagosomes while dynein, which has been suggested to be involved in the centripetal transport of endocytic organelles^3,11,27,28,30^, had a much stronger effect on the effective centripetal transport of the large phagosomes. Furthermore, we found that the spatial distribution of microtubules and dyneins around phagosomes could explain part of the observed transport characteristics.

In summary, our findings suggest that a simple size-dependent cellular sorting mechanism might exist that can support transport of large phagocytosed bacteria towards the nucleus to facilitate their digestion and that simultaneously can support outward transport of small bacterial fragments, such as for antigen presentation^3,24–26,31^.

## Results

### Particle opsonization

Phagocytic targets with diameters between 1 µm and 3 µm were generated by opsonizing polystyrene particles with IgG. The use of fluorescently labelled secondary antibodies against IgG showed that the opsonization was successful (Figure S1). Particles with IgG opsonization showed a strong fluorescence signal (Figure S1B), whereas particles without IgG showed no detectable signal (Figure S1C).

### Particle uptake

Particle uptake by macrophages was induced either by particle sedimentation onto the cells or by bringing the particles in contact with the cells using holographic optical traps^32,33^. The majority of the phagosome tracks were acquired by letting the particles sediment onto the cells to induce particle-cell binding. This method has the advantage of allowing the simultaneous tracking of a large number of phagosomes in multiple cells. However, it does not guarantee that the all phagosomal motion is recorded completely, starting with the beginning of the uptake process. To test whether the potentially missing first segments of the phagosomal tracks have an influence on the determined phagosomal transport characteristics, we performed additional experiments with full control over the starting time point of phagocytosis. For this purpose, we used holographic optical tweezers to move individual particles to the cell membrane of a macrophage to induce phagocytic uptake. We found that the determined transport characteristics do not depend on whether the particles were brought into contact with the cells by sedimentation or by the optical tweezers, and we therefore pooled the data for subsequent analysis.

We used fluorescently labelled secondary antibodies against IgG to measure whether particles that were brought into contact with the cell membrane were subsequently phagocytosed (Figure S2). The cells were chemically fixed 15 min, 30 min and 60 min after the particles were brought into contact with the cells. Subsequently, the fluorescent secondary antibody was added to the cell, which resulted in fluorescent labeling of the particles that were not internalized. We found that as early as after 15 min, more than 86 ± 11% of the particles that were in contact with the cells were internalized (Figure S2).

### General aspects of phagosomal transport

**Figure 2:**
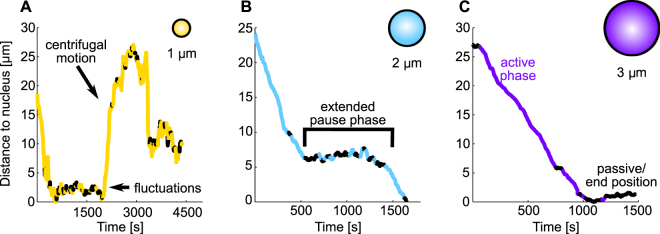
Examples of the phagosome trajectories for particles with different diameters. Shown are representative trajectories of particles with a diameter of 1 µm (**A**), 2 µm (**B**) and 3 µm (**C**). Plotted are the distances of the particle position to the nearest point of the nucleus as a function of time. The trajectories were subdivided into segments with a length of 30 seconds. The classification into segments of active (bright/colored trajectory parts) and passive motion (dark/black trajectory parts) was done as described in the Materials and Methods section. (**A**) Particles with a diameter of 1 µm typically had frequent phases of active centrifugal motion and frequent phases of passive fluctuations. (**B**) Particles with a diameter of 2 µm typically had extended pause phases that interrupted the active motion phases before the nucleus was reached. (**C**) Long persistent phases of active centripetal motion and passive phases after the nucleus was reached were typical for particles with a diameter of 3 µm. Frequent well-defined end positions close to the nucleus were observed only for the 2 µm and 3 µm particles, but not for the 1 µm particles.

We analyzed more than 50 phagosome tracks for every investigated particle size and found strong qualitative and quantitative differences between the transport characteristics of phagosomes of different sizes. Phagosomes with a diameter of 3 µm typically showed a highly persistent and efficient transport towards the nucleus, with only few extended pauses and very little motion towards the cell periphery. Figure 2C shows an example of a trajectory of such a phagosome. In contrast, intermediate-sized phagosomes with a diameter of 2 µm showed a strongly increased amount of extended pause phases that reduced the efficiency and the persistence of the transport (Fig. 2B shows a representative trajectory). Small phagosomes with a diameter of 1 µm, however, showed strongly increased phases of centrifugal motion, leading to a strongly reduced effective velocity (Fig. 2A shows a representative trajectory).

To quantify the observed size-dependent differences in phagosomal transport, we determined various parameters that characterized phagosomal transport behavior.

### Centrifugal transport of phagosomes

**Figure 3:**
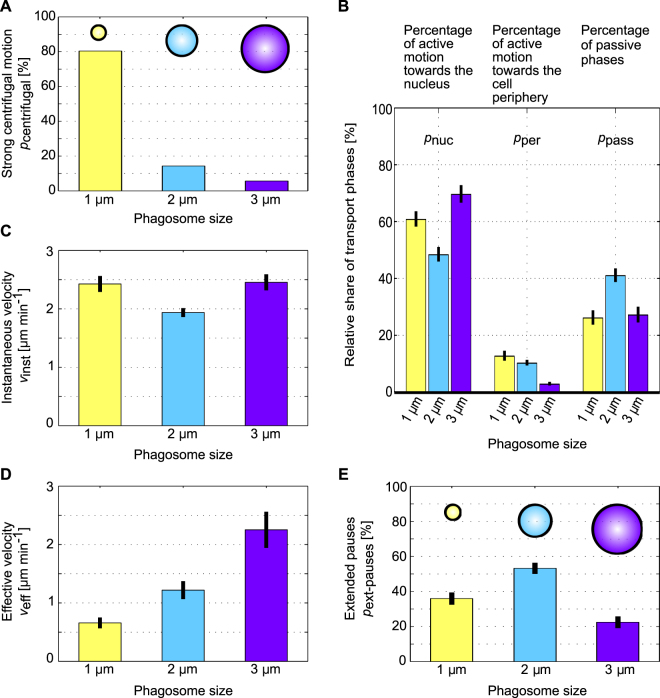
Size-dependent characteristics of phagosomal transport. (**A**) Percentage p_centrifugal_ of phagosomes that show a strong centrifugal motion towards the cell periphery after reaching the nucleus. Small phagosomes with a diameter of 1 µm show a strong centrifugal transport in 82% of cases, whereas medium- and large-sized phagosomes show such transport in only 17% and 6% of the cases, respectively. (**B**) Relative share of different phases of phagosomal transport. Phagosomes with a diameter of 3 µm show the highest percentage of active motion towards the nucleus, p_nuc_, and the smallest percentage of active motion towards the periphery, p_per_. Phagosomes with a diameter of 2 µm show the largest percentage of passive motion, p_pass_. Phagosomes with a diameter of 1 µm show the largest percentage of active motion towards the cell periphery. (**C**) Instantaneous transport velocities, v_inst_. These velocities are similar to each other for the three different phagosome sizes and range between 1.9 and 2.5 µm min^−1^. (**D**) Effective velocities of the whole active phagosome transport, v_eff_. These velocities strongly depend on the particle diameter and increase with the phagosome size. (**E**) Percentage of extensive pause phases, p_ext-pauses_. Particles with a diameter of 2 µm show the highest percentage of extended pauses. Error bars are the std. error of the mean. We analyzed 51, 63, and 53 trajectories for the phagosomes with 1 µm, 2 µm, and 3 µm diameters.

To quantify the frequency of strong centrifugal transport, we determined the percentage of phagosomes, *p*_centrifugal_, that leave the perinuclear region after reaching the nucleus. Phagosomes were considered to leave the perinuclear region when they were transported centrifugally by at least 5 µm after reaching the nucleus^10^. Figure 3A shows *p*_centrifugal_ for the three different phagosome sizes. Phagosomes with a diameter of 1 µm show a strong centrifugal transport after they have reached the nucleus in 82% of the cases, whereas phagosomes with diameters of 2 µm and 3 µm show such transport only in 17% and 6% of the cases, respectively.

### Distribution of transport phases

To analyze the percentages of active centripetal motion (towards the perinuclear region), active centrifugal motion (towards the cell periphery) and passive motion, we determined the values *p*_nuc_, *p*_per_, and *p*_pass_, respectively, as described in the Materials and Methods. Figure 3B shows that large phagosomes with a diameter of 3 µm exhibit the highest percentage of active motion towards the nucleus, *p*_nuc_ = 70 ± 3% (mean ± standard error of the mean), and the lowest percentage of active motion towards the periphery, *p*_per_ = 3 ± 1%. Medium-sized phagosomes with a diameter of 2 µm show the largest percentage of passive motion, *p*_pass_ = 41 ± 2%. Small phagosomes with a diameter of 1 µm show the largest percentage of active motion towards the cell periphery, *p*_per_ = 13 ± 2%.

The large share of active motion towards the nucleus in combination with the small share of motion towards the periphery leads to a very high efficiency of phagosomal transport towards the perinuclear region for the large 3 µm-sized particles. In contrast, the large share of directed motion towards the cell periphery leads to less efficient phagosomal transport for the small 1 µm-sized particles.

### Velocities of phagosomal transport

To further describe the efficiency of the transport process quantitatively, we determined the instantaneous velocity, *v*_inst_, and the effective velocity, *v*_eff_, of the transport for all three phagosome sizes, as described in the Materials and Methods. The instantaneous velocity *v*_inst_ is a measure of phagosomal transport over a short timescale of 30 seconds, whereas the effective velocity *v*_eff_ quantifies the efficiency of the transport on a longer timescale, typically several tens of minutes.

Figure 3C shows that the mean instantaneous velocity *v*_inst_ is similar for all phagosome sizes, ranging between 1.9 and 2.5 µm min^−1^. Nevertheless, the active movement of 2 µm-sized phagosomes is slightly slower (*v*_inst_ = 1.9 ± 0.1 µm min^−1^) than the active motion of the other phagosomes (*v*_inst_ = 2.4 ± 0.1 and 2.5 ± 0.1 µm min^−1^).

A closer look at the instantaneous velocities of every single active segment with respect to the transport direction provides additional insights into the size-dependent transport velocities. The distribution of the instantaneous velocities towards the nucleus and the distribution of the instantaneous velocities towards the cell periphery were only comparable for 1 µm particles (Figure S3). For larger phagosomes, the instantaneous velocities towards the nucleus were significantly larger than towards the periphery.

While the mean instantaneous velocities (Fig. 3C) were similar to each other for the three different phagosome sizes, the effective velocities *v*_eff_ showed a strong dependence on phagosome size (Fig. 3D). The effective velocity of 3 µm phagosomes, *v*_eff_ = 2.3 ± 0.3 µm min^−1^ (mean ± s.e.m.), was significantly larger than the effective velocities for the other two phagosome sizes, and it was nearly the same as the mean instantaneous velocity for the large phagosomes. This is expected for a largely unidirectional, persistent ballistic transport. For 2 µm-sized phagosomes, the effective velocity was significantly smaller, with a value of *v*_eff_ = 1.2 ± 0.2 µm min^−1^ due to the increased share of passive phases. In contrast, 1 µm phagosomes showed the lowest effective velocity, *v*_eff_ = 0.7 ± 0.1 µm min^−1^, due to frequent changes in the direction of the transport.

### Persistence of phagosome movement

**Figure 4:**
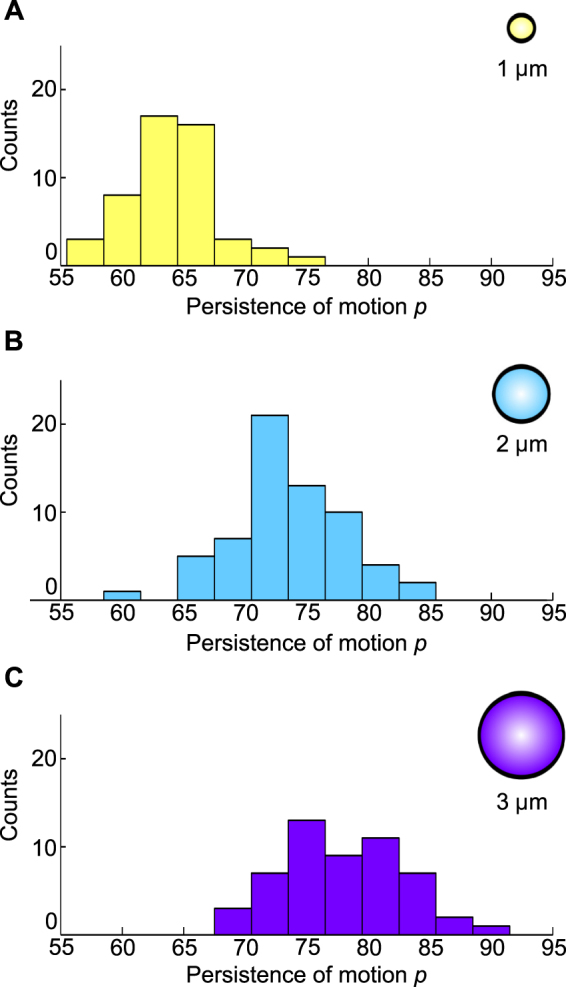
Persistence of phagosomal transport. Distributions of the persistence of the motion for phagosomes with a diameter of 1 µm (**A**), 2 µm (**B**), and 3 µm (**C**). The persistence of the transport increases with increasing phagosome diameter. We analyzed 51, 63, and 53 trajectories for the phagosomes with 1 µm, 2 µm, and 3 µm diameters.

To quantify changes in the direction of active motion, we determined the persistence *p* of the phagosomal transport for the three different organelle sizes, as described in the Materials and Methods. We found that the persistence of the motion *p* was strongly dependent on phagosome size (Fig. 4). The transport of 3 µm particles showed the highest persistence, with a mean value of *p* = 78 ± 1% (mean ± s.e.m.), in agreement with the highest effective velocity as well as the highest percentage of transport phases towards the nucleus. Phagosomes with a diameter of 2 µm exhibited a smaller mean persistence of *p* = 74 ± 1%, and 1 µm-sized phagosomes showed the lowest mean persistence with a value of *p* = 64 ± 1%, due to frequent changes in the active transport direction. This low persistence of 1 µm phagosomes agrees with the nearly equal distribution of instantaneous velocities in both directions (Figure S3).

### Extended pause phases

We noticed that 2 µm phagosomes often showed extensive pauses during their transport from the cell periphery towards the perinuclear region. To quantify this behavior, we determined the percentage of extensive pause phases, *p*_ext-pause_, as described in the Materials and Methods for the three different phagosome sizes (Fig. 3E).

We found that the medium-sized 2 µm particles exhibit the highest percentage of extensive pause phases, *p*_ext-pause_ = 53 ± 3% (mean ± s.e.m.), whereas the other two phagosome sizes show less extensive pauses, with *p*_ext-pause_ = 22 ± 3% for the 3 µm-sized particles and *p*_ext-pause_ = 36 ± 3% for the 1 µm-sized particles.

### Influence of actin filaments

To investigate the molecular basis for the observed size-dependent transport behavior, we studied the influence of actin-based motility on phagosomal transport. The modes in which actin filaments can be involved in intracellular transport processes are very versatile: the filaments can act as tracks for myosin motors that move organelles, they can drive organelle motion by exerting polymerization forces or they can be involved in transport processes by allowing objects to couple to their retrograde flow^11,28,32,34^.

**Figure 5:**
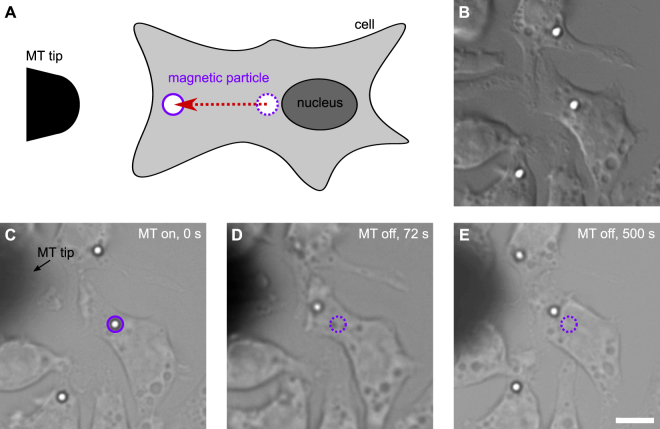
Magnetic tweezers experiments with cytochalasin D-treated cells. (**A**) Sketch of the experiment (not drawn to scale). The magnetic tweezers tip is positioned close to J774 cells with internalized superparamagnetic particles and switched on to move the phagosomes from the perinuclear region to the cell periphery. (**B**)–(**E**) Time series of an experiment. (**B**) DIC image of J447 cells with internalized 2.8 µm-sized IgG-coated Dynabeads. (**C**) The tip of the magnetic tweezers was moved close to the cells and the magnetic tweezers were turned on (t = 0 s). (**D**) After 72 s, the phagosome, which is labeled by the solid violet circle in panel (C), has moved towards the magnetic tweezers tip in the cell periphery and the magnetic tweezers were turned off. (**E**) The subsequent phagosomal transport back into the direction of the perinuclear region was tracked. The original location of the phagosome in panel (C) is indicated by the dashed violet circle in panel (D) and (E). The scale bar is 10 µm.

We investigated the influence of actin filaments on size-dependent phagosomal transport by using the actin depolymerizing drug cytochalasin D (cytoD)^35^ in a magnetic tweezers-based transport assay, as described in the Material and Methods. Briefly, we allowed the cells to phagocytose superparamagnetic particles before we treated them with 2 µM cytoD, since the actin cytoskeleton is required for the phagocytic uptake^36^. The particles were coated with IgG and had diameters of 1 µm and 2.8 µm. Due to the effective and persistent transport of the large 2.8 µm-sized phagosomes, the majority of these organelles had already finished their movement to the perinuclear region after the cytoD treatment was finished. To investigate the phagosomal transport nevertheless, we used magnetic tweezers^37^ to pull the particles back to the cell periphery. After switching off the tweezers, we tracked the subsequent phagosomal motion (Fig. 5). We applied this measurement protocol in experiments with the large (2.8 µm) and small (1 µm) superparamagnetic particles.

**Figure 6:**
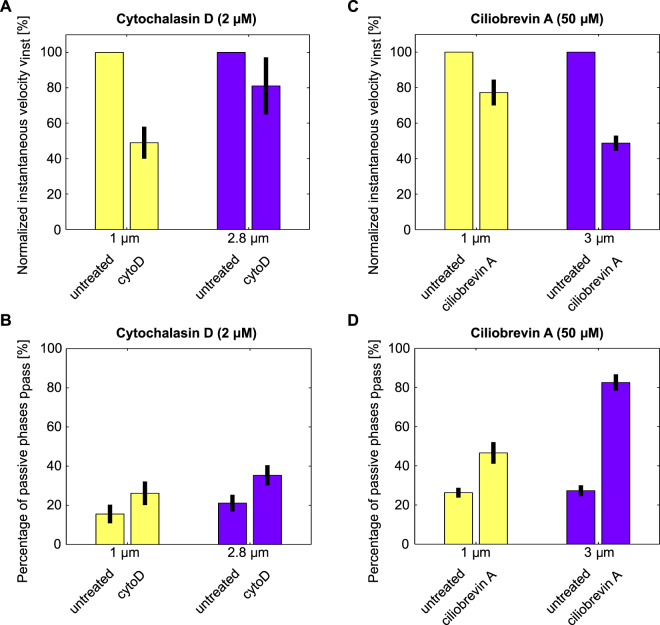
Influence of cytochalasin D (cytoD) and ciliobrevin A on phagosomal transport. (**A**) Normalized instantaneous velocities, v_inst_, for untreated cells and cells treated with 2 µM cytoD. For small 1 µm-sized phagosomes, the velocity was reduced to 49 ± 9% in cytoD-treated cells compared to untreated cells. For the large phagosomes with a diameter of 2.8 µm, the velocity was decreased to 81 ± 16%. (**B**) Percentage of passive phases, p_pass_, for untreated cells and cells treated with 2 µM cytoD. For the small phagosomes, p_pass_ was increased from 16 ± 5% to 26 ± 6% in cytoD-treated cells compared to untreated cells, whereas for the large phagosomes, it was increased from 21 ± 4% to 35 ± 5%. (**C**) Normalized instantaneous velocities for untreated cells and cells treated with 50 µM ciliobrevin A. For the small phagosomes, v_inst_ was decreased to 77 ± 7% in ciliobrevin A-treated cells compared to untreated cells, whereas it was decreased to 49 ± 4% for the large phagosomes. (**D**) Percentage of passive phases for untreated cells and cells treated with 50 µM ciliobrevin A. For the small phagosomes, p_pass_ was increased from 26 ± 3% to 47 ± 6% in ciliobrevin A-treated cells compared to untreated cells, whereas for the large phagosomes, it was increased from 27 ± 3% to 83 ± 4%. The error bars show the standard error of the mean. To investigate the influence of cytoD, we analyzed 16 and 21 trajectories for the small and large phagosomes in untreated cells, and 16 and 22 trajectories for the small and large phagosomes in cytoD-treated cells. To investigate the influence of ciliobrevin A, we analyzed 51 and 53 trajectories for the small and large phagosomes in untreated cells, and 15 trajectories for the small as well as the large phagosomes in ciliobrevin A-treated cells.

We found that the cells treated with cytoD showed a reduced instantaneous phagosomal velocity compared to the untreated cells for small phagosomes and a barely changed velocity for large phagosomes (Fig. 6A). For the small 1 µm-sized phagosomes, the velocity was decreased by 51 ± 9% (mean and std. error of mean), whereas for the large 2.8 µm-sized organelles, it was decreased by only 19 ± 16%. Furthermore, we found that the transport in cells treated with cytoD showed an increased percentage of passive phases, *p*_pass_, compared to the untreated cells for large and small phagosomes (Fig. 6B). In both cases, *p*_pass_ was increased by a factor of 1.7.

### Inhibition of dynein motors

To further investigate the molecular basis for the observed size-dependent transport behavior, we studied the influence of dynein-based motility on the phagosomal transport. We inhibited dynein with 50 µM ciliobrevin A, which reduces the ATPase activity of the motor^38^, and we measured the phagosomal transport characteristics as described. We found that the inhibition of dynein changes the phagosomal transport in a size-dependent manner. Small 1 µm-sized phagosomes as well as large 3 µm-sized phagosomes showed a reduced instantaneous velocity in ciliobrevin A-treated cells compared to untreated cells. However, the effect of the ciliobrevin A treatment was stronger for the large organelles: in the case of the small phagosomes, the velocity was decreased by 23 ± 7%, whereas for the large phagosomes it was even decreased by 51 ± 4% (Fig. 6C). In addition, the percentage of passive phases was increased in ciliobrevin A-treated cells compared to untreated cells. The effect of the ciliobrevin A treatment was also stronger for the larger phagosomes: in the case of the small organelles, the percentage of passive phases was increased by 21 ± 7%, whereas for the large organelles, it was increased by 56 ± 5% (Fig. 6D).

In addition, we found that for all phagosome sizes the percentage of active motion towards the nucleus was decreased in the ciliobrevin A-treated cells (Figure S4A) compared to the untreated cells (Fig. 3B). However, these changes were much stronger in the case of the large 3 µm phagosomes than in the case of the small and intermediate-sized phagosomes with 1 µm and 2 µm diameters, respectively.

The strong inhibition of the centripetal transport of the large phagosomes can also be seen if the percentage of phagosomes that reached the nucleus is determined in treated and untreated cells. Treatment with ciliobrevin A reduced this percentage by 51% in the case of 3 µm phagosomes (Figure S4B). Furthermore, the percentage of extended pauses of 3 µm phagosomes increased strongly in the treated cells (Figure S4D) compared to the untreated cells (Fig. 3E).

While small phagosomes show a large amount of strong centrifugal motion in untreated and ciliobrevin A-treated cells, intermediate-sized phagosomes only show a large amount of this motion after ciliobrevin A treatment (Figure S4C and Fig. 3A).

**Figure 7:**
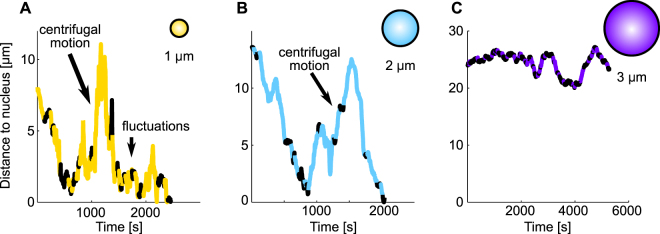
Examples of phagosome trajectories in cells treated with the dynein motor inhibitor ciliobrevin A for phagosomes with different diameters. Shown are representative trajectories for phagosomes with a diameter of 1 µm (**A**), 2 µm (**B**) and 3 µm (**C**), analogous to the data shown in Fig. 2. The trajectories for the small 1 µm phagosome (**A**) and the intermediate-sized 2 µm phagosome (**B**) show various characteristic features, such as a strong centrifugal motion after the nucleus was reached. The trajectory for the large 3 µm phagosome (**C**) shows a characteristically large amount of motion towards the periphery and no reaching of the nucleus.

Representative examples of phagosome trajectories that show several of the abovementioned characteristics are displayed in Fig. 7.

### Distribution of dynein motors

**Figure 8:**
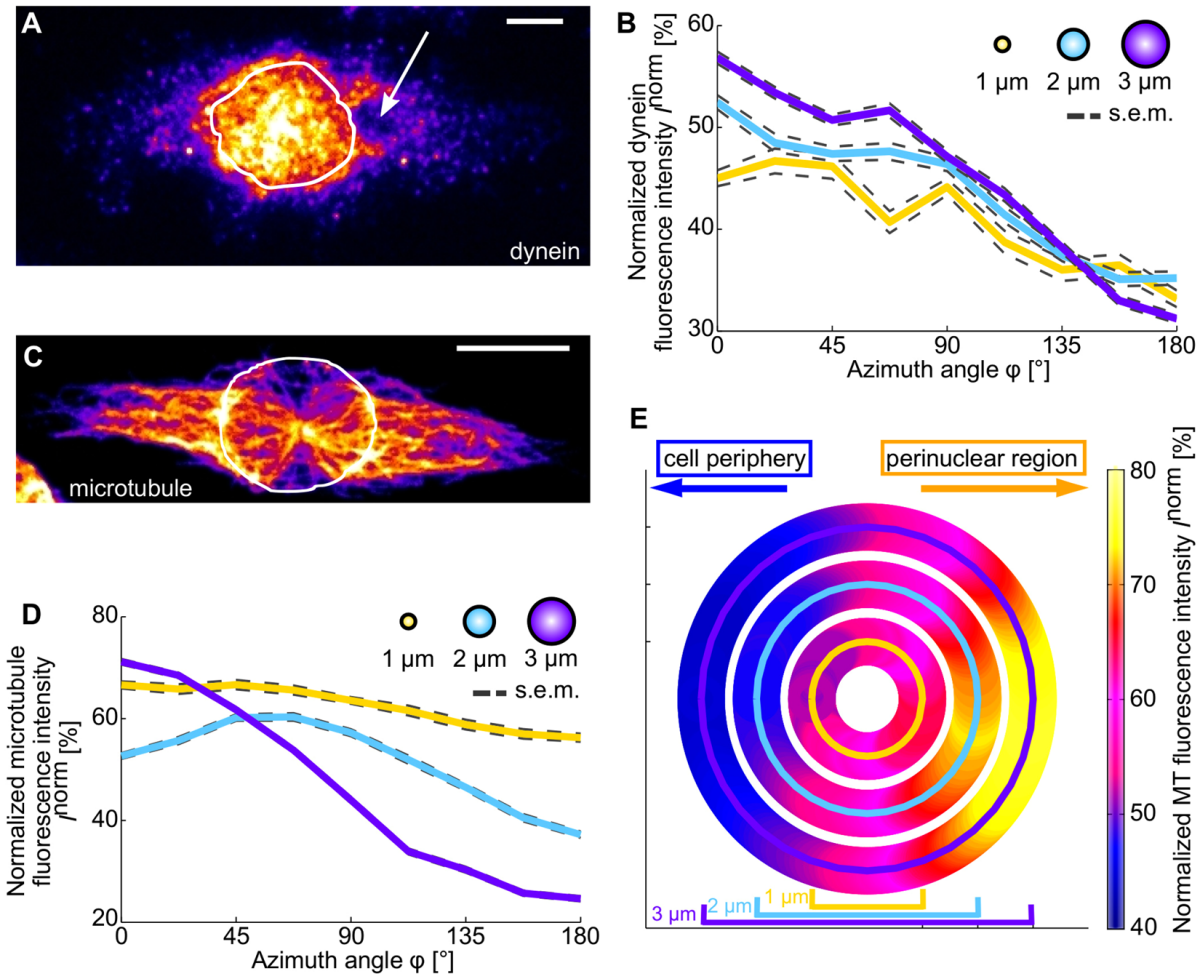
Distribution of dyneins and microtubules in macrophages. (**A**) Distribution of dynein motor proteins inside the cell and around a phagosome (white arrow) in a J774 mouse macrophage (false color image, average intensity projection of image stack). The intermediate chain of cytoplasmic dynein was labelled with a secondary antibody by immunostaining. The dynein distribution shows clustering within the cytoplasm and around the phagosome. The border of the nucleus is visualized by the white line. The scale bar is 5 µm. (**B**) Normalized intensity distributions of labelled dynein motors around phagosomes with diameters of 1 µm, 2 µm, and 3 µm as a function of the azimuth angle φ (φ = 0° represents the direction towards the nucleus, φ = 180° represents the direction towards the cell periphery). The results represent more than 50 analyzed phagosomes for every phagosome size. (**C**) Distribution of microtubules in a J774 mouse macrophage (not the same cell as shown in panel (A)) without phagosomes (false color image, average intensity projection of image stack). Alpha-tubulin was labelled with secondary antibody by immunostaining. The bright spot close to the center of the cell is the MTOC. The microtubule density is larger in the perinuclear region compared to the cell periphery. The border of the nucleus is visualized by the white line. The scale bar is 10 µm. (**D**) Normalized intensity distributions of labelled microtubules around phagosomes with diameters of 1 µm, 2 µm, and 3 µm as a function of the azimuth angle φ. The results represent more than 50 analyzed phagosomes for every phagosome size. (**E**) Polar plots of microtubule densities around spheres of different sizes within macrophages. The microtubule densities within the cells were measured by confocal fluorescence microscopy. Displayed are the normalized intensity distributions of fluorescently labelled microtubules around the circumference of spheres with diameters of 1, 2 and 3 µm, representing phagosomes of these different sizes. The results represent the average intensity distributions of 343 analyzed spheres for every particle size.

Since the dynein inhibitor ciliobrevin A had a size-dependent influence on the phagosomal transport characteristics, we measured the distribution of the dynein motors around phagosomes of different sizes within the cells by immunofluorescence spinning disk confocal microscopy, as described in the Materials and Methods. A representative image of a cell with labelled dyneins is displayed in Fig. 8A. The dynein motors show clustering within the cytoplasm and around the phagosome. The density of dyneins in the region around the nucleus is larger than in the perinuclear region. Consequencly, phagosomes exhibit a larger amount of dyneins on their nucleus-facing side compared to their cell periphery-facing side. This asymmetry increases with increasing phagosome size (Fig. 8B). For large 3 µm-sized phagosomes, the dynein density on the nucleus-facing side was approximately Δ*I*_dynein_ = 20% higher than on the periphery-facing side. For intermediate-sized spheres with a diameter of 2 µm, this difference was 14%, and for small 1 µm-sized spheres the difference was 11%. The data shown in Fig. 8B is based on fluorescence intensity distributions that were normalized for each phagosome (as described in the Materials and Methods) to compensate for random variations in the fluorescence labelling efficiency. Therefore, the data shows relative differences in the amount of dyneins around phagosomes. However the data does not show that more motors can be associated with larger phagosomes compared to smaller phagosomes because of their larger surface area.

### Distribution of microtubules

Since microtubules are the basis of dynein-driven phagosomal transport, we also investigated the microtubule (MT) distribution inside macrophages and around phagosomes of different sizes.

We measured the MT distribution in macrophages with and without phagosomes by using immunofluorescence spinning disk confocal microscopy, as described in the Materials and Methods. A representative image of the MT distribution in a macrophage without phagosomes is shown in Fig. 8C. The density of the MTs is generally higher in the perinuclear region, close to the microtubule organizing center (MTOC), than in the cell periphery.

Given this MT gradient inside a cell, phagosomes placed in the cell would typically be confronted with a larger MT density at their nucleus-facing side compared to their periphery-facing side. This asymmetry between the different sides of the phagosome increases with increasing phagosome size (Fig. 8E). For large spheres with a diameter of 3 µm, the MT density on the nucleus-facing side was approximately Δ*I*_MT-spheres_ = 29% higher than on the periphery-facing side. For intermediate-sized spheres with a diameter of 2 µm, this difference was 22%, and for small 1 µm-sized spheres the difference was 12%.

Next, we tested whether this size-dependent asymmetry in the MT distribution is also present around real phagosomes inside macrophages. We measured the distribution of MT around phagosomes of various sizes, analogously to the measurement of the dynein distributions around phagosomes. We found that phagosomes of all sizes were confronted with a larger MT density at their nucleus-facing side compared to their cell periphery-facing side (Fig. 8D). However, this asymmetry increased with increasing phagosome size. For the large phagosomes with a diameter of 3 µm, the MT density at the nucleus-facing side was approximately Δ*I*_MT-phagosomes_ = 40% larger than at the periphery-facing side. For intermediate-sized phagosomes with 2 µm diameter, this difference was 15%, and for small phagosomes with a size of 1 µm this density difference was only approximately 9%. As in the cased of the dynein motor distributions (Fig. 8B), the data shown in Fig. 8D is based on normalized intensity distributions (as described in the Materials and Methods). Therefore, the data shows relative differences in the amount of MT around phagosomes. However the data does not show that more MT can associate with larger phagosomes compared to smaller phagosomes because of their larger surface area.

## Discussion

We found that phagosomal transport in macrophages depends strongly on phagosome size in a qualitative and quantitative manner. Large phagosomes with a diameter of 3 µm were transported with the largest persistence and the largest effective velocity towards the nucleus. This was due to the largest share of active motion towards the nucleus and the smallest share of active motion towards the cell periphery. After the phagosomes reached the nucleus, they exhibited a very low amount of strong centrifugal transport. Medium-sized phagosomes with a diameter of 2 µm were transported with an intermediate effective velocity and an intermediate persistence. Furthermore, their transport was characterized by the largest amount of extended pause phases. After these phagosomes reached the nucleus, they also exhibited a low amount of strong centrifugal transport. In contrast, small phagosomes with a diameter of 1 µm were transported with the lowest persistence and the lowest effective velocity towards the nucleus. This was due to the largest percentage of motion towards the periphery and the highest amount of strong centrifugal transport. Because of the strong centrifugal transport, a well-defined transportation end-point in the perinuclear region was missing for this phagosome size.

To investigate the molecular basis for this size-dependent phagosomal transport behavior, we studied the influence of actin-based motility on the transport. Actin has been suggested to be involved in phagosomal transport, particularly in transport on short length scales^11,28^. In general, actin-based motility can for example be caused directly by actin-binding molecular motors, such as myosins, or by coupling to the retrograde flow of actin. The instantaneous velocities that we observed for all three phagosome sizes were in the range of approximately 30 to 40 nm s^−1^ (1.9 to 2.5 µm min^−1^), which is comparable to the speed of retrograde actin flow^39,40^. We used cytochalasin D to depolymerize actin filaments and found that actin-based motility is involved in the transport of both large and small phagosomes. However, it had a stronger effect on the transport of the small phagosomes. For the large phagosomes, actin-based motility seems to not be the dominating mechanism for the observed transport behavior.

We therefore also investigated the influence of microtubule-based motility driven by the molecular motor dynein. We used ciliobrevin A to inhibit dynein, which was suggested to be involved in the centripetal transport of endocytic organelles^3,11,27,28^. We found that dynein is important for the centripetal transport of phagosomes of all sizes. However, it had the strongest effect on the persistent effective transport of the large phagosomes. After treatment with ciliobrevin A, the instantaneous velocity of the large phagosomes decreased by approximately 51 ± 4%. In addition, the percentage of active motion towards the nucleus decreased from 70 ± 3% in untreated cells to 13 ± 4% in ciliobrevin A-treated cells, and the percentage of passive phases increased from 27 ± 3% to 83 ± 4%. Furthermore, the percentage of phagosomes that reach the nucleus decreased from 91% to 40%. These observations indicate that although they are involved in the transport of phagosomes of all the investigated sizes, dyneins are particularly important for the transport of large phagosomes. Actin-based motility, in contrast, seems to play a larger role in the transport of small phagosomes.

These findings raise the question of how phagosome size could influence the involvement of different transport mechanisms. This could occur in general by currently unknown phagosome size-dependent biochemical signaling pathways. However, our findings suggest that the architecture of the intracellular microtubule network might also plays a role by supporting a size-dependent involvement of dyneins. We found that phagosomes of all sizes are confronted with a larger microtubule and dynein density on their nucleus-facing side compared to their cell periphery-facing side. Furthermore, we show that this asymmetry increases with increasing phagosome size. These finding suggest that large phagosomes in particular exhibit a much higher density of dyneins at their nucleus-facing side compared to their periphery-facing side. A higher density of dyneins could increase the probability of dynein cluster formation. Clustering of dyneins on phagosomes has recently been observed on the surface of isolated phagosomes by Rai and colleagues. These clusters were shown to be important for phagosomal transport and maturation^41^. In contrast to kinesins, which are mainly involved in centrifugal organelle transport, dyneins have been shown to act in teams to generate large collective forces^42^. Our findings therefore let us hypothesize that dynein clustering on large phagosomes allows the generation of large collective forces directed towards the nucleus, leading to a persistent phagosomal transport in centripetal direction and potentially to a faster maturation.

In contrast to the large phagosomes, small phagosomes might show less clustering of dyneins leading to less cooperative dynein activity. Such a lower dynein activity would allow actin-based motility, in form of motor activity or coupling to retrograde flow or motility based on other motors such as kinesin to contribute more significantly to phagosomal transport, leading to the more bidirectional transport observed here.

The instantaneous transport velocities observed in this study were on the order of approximately 30 nm s^−1^. This velocity is much smaller than the velocities of dynein motors observed *in vitro*, which ranged between 200 and 700 nm s^−1^ ^43–45^. However, drag forces inside cells, due to the crowded intracellular environment, are expected to slow down transport based on dynein or other molecular motors. The transport velocities that we found here agree with reported velocities of microtubule-based phagosomal transport inside cells, which range between 25 and 100 nm s^−1^ ^10,18,46^.

The centrifugal transport of phagosomes with a diameter of 1 µm has previously been described by Zhang *et al*.^26^. However, in contrast to their suggestion that centrifugal transport is a property of all phagosomes, we show here that centrifugal transport is strongly reduced for larger phagosomes. This size-dependent transport back to the cell periphery could potentially assist important cellular functions, since it would support the exocytosis of phagosomal organelles, such as phagolysosomes containing indigestible material, so called “residual bodies” or vesicles containing debris of the digestion process or antigens^26^.

In summary, our results show that phagosomal transport in general and transport efficiency towards the nucleus in particular are strongly influenced by the size of the phagosome. We found that large phagosomes exhibit a persistent centripetal transport whereas small phagosomes show strongly bidirectional motion.

Furthermore, we show that the transport of large phagosomes is largely driven by dyneins whereas the motion of small phagosomes is largely based on actin-filaments. In addition, we found that also the microtubule and dynein distributions around phagosomes depend on the organelle size and that the asymmetry between the nucleus-facing side and the opposite side increases with increasing phagosome size. We hypothesize that in addition to so far unknown phagosome size-dependent biochemical signaling, these differences might explain part of the discovered size-dependent transport characteristics.

Our findings regarding the size-dependent phagosomal transport and its possible underlying mechanisms might contribute to optimizing particle-based drug carriers that are targeted to either the perinuclear region or the cell periphery. Furthermore, our findings suggest that a basic size-dependent cellular sorting mechanism might exist that increases centripetal transport of large phagocytosed bacteria to support their digestion, and that simultaneously increases centrifugal transport of small bacterial fragments, such as in antigen presentation. If such a size-dependent intracellular sorting leads to faster maturation and degradation of larger phagosomes, drugs that cause a clustering of pathogens could potentially lead to more efficient clearing by macrophages.

## Materials and Methods

### Experimental Design

#### Cell culture

Murine macrophage J-774A.1 cells (generous gift from Maximiliano Gutierrez, The Francis Crick Institute, London and purchased from DSMZ, Braunschweig, Germany) were cultured in Dulbecco’s Modified Eagles Medium (DMEM, containing 4.5 mM glucose, GIBCO, Carlsbad, California) supplemented with 10% heat-inactivated fetal bovine serum (BIOCHROM, Berlin, Germany) and 5% 2 mM L-glutamine (THERMO SCIENTIFIC, Braunschweig, Germany) under standard culture conditions (37 °C, 5% CO_2_, humidified). For imaging, cells were washed with Dulbecco’s Phosphate Buffered Saline (DPBS, GIBCO, Carlsbad, California), scraped off T-25 culture flasks (CORNING, Corning, New York) and collected by centrifugation (200 g, 2 min, 20 °C). The supernatant was removed, and the cell pellet was resuspended in 5 ml culture medium. Cells were seeded on microscope cover glasses (diameter: 18 mm, #1, MENZEL GLAESER, Braunschweig, Germany) in 12-well plates (Cellstar, GREINER BIO-ONE, Frickenhausen, Germany) containing 50–75 µl cell suspension and 1 ml culture medium, for 24–48 h under standard culture conditions.

#### Particles

White carboxylated polystyrene microspheres with diameters of 1 µm, 2 µm and 3 µm (MICROMOD, Rostock, Germany) were used as phagocytic targets. Native immunoglobulin G (IgG) primary antibodies from mouse serum (MERCK MILLIPORE, Darmstadt, Germany) were passively adsorbed onto the particles by the following procedure: Microspheres were washed with MES buffer (2-(*N*-morpholino)ethanesulfonic acid, 25 mM, pH 6, SIGMA-ALDRICH, St. Louis, Missouri) three times, and incubated in 1 ml of 0.5 mg ml^−1^ mouse IgG in MES buffer overnight to achieve opsonization. The beads were washed three times with DPBS (centrifugation parameters: 2000 g, 20 min, 20 °C) to remove unbound IgG. After centrifugation, the pellet was resuspended in PBS (0.1 M, pH 7.2, LONZA, Basel, Swiss, containing 0.1% glycine) at the desired concentration for storage.

Opsonization was verified by antibody-antibody labeling with goat anti-Mouse IgG cross-adsorbed fluorescent secondary antibody (Dy-Light 488, THERMO SCIENTIFIC). For cell experiments, IgG-opsonized particles were diluted in image medium (Minimum Essential Media (MEM, GIBCO) containing 5% 1 M HEPES (GIBCO) and 1% Penicillin-Streptomycin (GIBCO, 10000 Units ml^−1^ Penicilin, 10000 µg ml^−1^ Streptomycin)).

#### Induced phagocytosis

Particle engulfment was induced either by particle sedimentation onto the cells or by bringing the particles into contact with the cells by using holographic optical tweezers^47^. For the generation of multiple optical traps, a 1064 nm fiber laser (IPG YLM-5-LP-SC, max. output 5.6 W, IPG PHOTONICS, Oxford, Massachusetts) was used. The laser was stabilized by a noise eater feedback loop (acousto optic modulator (AOM): AA Optoelectronic, photo diode and driver: TEM MESSTECHNIK, Hannover, Germany) and split into multiple foci by a spatial light modulator (SLM, XY Phase Series, BOULDER NONLINEAR SYSTEMS, Lafayette, Colorado). Controlled particle positioning in the sample was done by a phase modulation of the laser by the SLM display via a custom-made MATLAB and LABVIEW program (MATLAB, Mathworks, Natick, Massachusetts and LABVIEW, National Instruments, Austin, Texas). The computer-generated holograms for the phase mask were calculated via complex addition of Fresnel lenses and blaze gratings^48^ and were displayed on the SLM display. The phase-modulated laser beam in the SLM plane was imaged onto the back-focal-plane of the microscope objective, resulting in the desired multi-foci trapping geometry in the focal plane of the microscope^32,33,49^.

#### Live cell imaging and optical trapping

Life cell imaging was performed at a sample temperature of 37 °C, which was enabled by a custom-made incubation chamber that encloses the microscope body. The volume of the chamber (~0.2 m^3^) was heated with a heating coil (NK-100-0,8-1, VENTS, Litvinov, Czech Republic), and a fan (KSA100-2E, VENTS) which was mechanically coupled only weakly to the microscope setup. The temperature in the chamber was measured with a PT100 resistor next to the sample and controlled with the heating coil via a PID (CN7500, NEWPORT ELECTRONICS, Santa Ana, California) feedback loop, resulting in a standard deviation of the temperature of 0.2 °C.

Cover slips with adherent cells were mounted into custom-made aluminum sample holders. For experiments based on particle sedimentation, particle solutions with approximately 10^8^ beads per ml were added. Bright field, DIC (differential interference contrast) and fluorescent imaging of the samples were realized using an inverted microscope (Nikon Eclipse Ti, NIKON, Tokyo, Japan) with high numerical aperture (NA) water immersion objectives (Lambda-S Series, Nikon, CFI Apo LWD 40x: NA = 1.15, CFI Plan Apo IR 60x: NA = 1.27). Image series were acquired with an EM-CCD camera (Luca-R, ANDOR, Belfast, Northern Ireland) at a frame rate of 0.5–2 Hz. For the tracking of 1 µm particles, it was necessary to acquire images at three different image planes with an axial distance of 1 µm at a 0.2 Hz frame rate to compensate for particle motion out of focus.

#### Magnetic tweezers experiments with cytochalasin D treatment

The magnetic tweezers experiments were done using an inverted light microscope (Nikon Eclipse Ti-U, Nikon, Tokyo, Japan) with a 60x magnification objective (CFI Plan Apochromat λ 60x oil objective, NA 1.40, NIKON). Image sequences were acquired with a CMOS camera (Orca-flash 4.0 v2, HAMAMATSU, Shizuoka, Japan) under bright field illumination. Cell condition and cell shape were monitored before and after the experiments by DIC imaging. The magnetic tweezers system consists of a solenoid with a high permeability soft iron core and a power supply with a maximum output power of 10 A (ELEKTRO AUTOMATIK, Viersen, Germany), as described previously^37^.

Superparamagnetic particles with a diameter of d = 1 μm (Dynabeads MyOne™, THERMO FISHER, Waltham, USA) and d = 2.8 μm (Dynabeads M-270, THERMO FISHER) were surface-functionalized as described for the carboxylated polystyrene microspheres above, added to the cell samples and left for 30 min for sedimentation and phagocytosis under standard culture conditions. Samples were washed and mounted into a custom-made heating-chamber suitable for magnetic tweezers measurements at a sample temperature of 37 °C. Subsequently, cytochalasin D (cytoD, C8273, SIGMA ALDRICH) drug treatment of cells was performed using a cytoD concentration of 2 μmol l^−1^ in image medium.

Magnetic particles in the proximity of the cell nucleus were identified, and the sample was positioned such that the lateral distance between the particles and the tip of the tweezers was approximately 20–30 μm. The height of the tweezers tip above the cover glass sample surface was approximately 30 µm. The particles were displaced by magnetic forces from the perinuclear region towards the cell periphery by using a solenoid current of 0.1 A and a voltage of 0.7 V. After sufficiently large displacement, the magnetic tweezers were switched off and consecutive phagosome transport behavior was monitored and analyzed as described above (Fig. 5).

#### Ciliobrevin A treatment

Cells were seeded on microscope cover glasses for 24–48 h under standard culture conditions. Ciliobrevin A (HPI-4, SIGMA-ALDRICH) was diluted in dimethyl sulfoxide (DMSO, SIGMA-ALDRICH) at a concentration of 10 mM and stored at −20 °C. Live cell microscopy experiments were performed at a ciliobrevin A concentration of 50 µM^50^. Before the experiments, the culture medium was removed from the cells, the ciliobrevin A diluted in image medium was added and the cells were incubated at 37 °C for 30 min. Just before the microscopy experiments started, cover slips with adherent cells were mounted into custom-made aluminum sample holders, and a solution of ciliobrevin A, image medium and particles was added.

Phagosomal uptake of particles was initiated by particle sedimentation and by the use of holographic optical traps as described above. Image acquisition, particle tracking and phagosomal trajectory analysis was performed as described above.

#### Dynein motor distribution

To label dynein motor proteins fluorescently, cells were first incubated for 24 h on cover glasses. Then, particle solutions with a concentration of approximately 10^8^ beads ml^−1^ in imaging medium were added and left for sedimentation and subsequent phagocytic uptake at 37° for 30–60 min. To avoid any influence of the size-dependent sedimentation time of the different sized particles, cells were stored at 4 °C for particle sedimentation before incubation at 37 °C. Cells were washed three times with DPBS to remove unattached particles and fixed with a PBS-paraformaldehyde (PFA) solution containing 4% PFA (SIGMA-ALDRICH, St. Louis, Missouri) on ice for 15 min. Afterwards, cells were washed again three times with DPBS, and a blocking buffer (94.7% PBS, including 0.3% Triton, SIGMA-ALDRICH, St. Louis, Missouri, and 5% goat serum, CELL SIGNALING, Cambridge, United Kingdom) was added for 60 min. After removing the blocking buffer, cells were incubated in a diluted primary antibody solution (1:200 DYNC1l2 rabbit antibody, THERMO SCIENTIFIC, in dilution buffer, consisting of 98.7% PBS, including 0.3% Triton and 1% BSA, APPLICHEM, Darmstadt, Germany) overnight by gentle mixing at 4 °C. Cells were then washed three times with DPBS and incubated in a diluted secondary antibody solution (1:100 anti-rabbit IgG (H + L), Alexa Fluor 488, THERMO SCIENTIFIC, in dilution buffer) for 2 hours at room temperature in the dark. Cells were washed again three times with DPBS and incubated in a dilute DNA-staining dye solution (1:1000 Hoechst 33342, CAYMAN, Ann Arbor, Michigan, in dilution buffer) for 2 minutes at room temperature in the dark. Cells were washed again three times with DPBS. Finally, the cover glasses were attached to a glass object holder (SERVOPRAX, Wesel, Germany) with Fluoromount-G (SOUTHERNBIOTECH, Birmingham, Alabama).

Z-stacks of labeled dynein were acquired on a DMI 6000 microscope (LEICA, Wetzlar, Germany, HCX PL APO 100x/1.40 OIL objective) including a spinning disk unit (CSU-X1, YOKOGAWA, Musashino, Japan) with an EMCCD camera (Evolve 512, PHOTOMETRICS, Tucson, Arizona, including an additional 1.2x magnification lens). To excite the secondary antibody, a 488 nm laser (50 mW, Sapphire 488, COHERENT, Santa Clara, California) was used at a spinning disk speed of 5000 rpm. To excite the Hoechst-labelled nucleus, a 405 nm laser (100 mW, Cube 405–100 C, COHERENT) was used at the same spinning disk speed. Axial stacks of multiple fluorescence images of the cells were acquired with a vertical distance of 0.25 µm, which was sufficient to oversample the image given the axial resolution of the microscope^51^. Bright field and DIC images were acquired within the focus plane of the phagosomes.

#### Microtubule distribution measurement

To label microtubules fluorescently, cells were prepared analogously to the preparation described in *Dynein motor distribution*. For additional measurements of microtubule distributions in cells without real phagosomes, fixed cell samples were prepared without phagocytosed particles. The protocol for the antibody-antibody labelling of the microtubules was identical to the protocol for dynein labelling. The primary antibody was α-tubulin (DM1A) mouse antibody (1:100–1:1000, CELL SIGNALING, Cambridge, United Kingdom), and the secondary antibody was anti-mouse (H + L) IgG (1:100–1:200 DyLight 488, THERMO FISHER or Alexa Fluor 594, CELL SIGNALING).

Z-stacks of labeled microtubules were acquired on the spinning disk microscope as described above. To excite the secondary antibody, a 488 nm (described above) and a 561 nm laser (50 mW, Saphire 561, COHERENT) was used at a spinning disk speed of 5000 rpm. To excite the Hoechst labelled nucleus, the 405 nm laser (described above) was used at the same spinning disk speed. Axial stacks of multiple fluorescence images of the cells were acquired with a vertical distance of 0.25 µm.

### Statistical Analysis

#### Intracellular particle tracking

Particles were tracked using a custom-written MATLAB cross-correlation algorithm providing a sub-pixel tracking precision with a position uncertainty of less than 6 nm. Figure 1 shows one frame of a DIC time lapse image series of a cell with two phagocytosed particles with overlaid particle trajectories. Absolute values of the particle positions for each image were transformed to polar coordinates with the origin manually set to the center of the cell nucleus, minus the radii of the particle and the nucleus.

#### Phagosomal trajectory analysis

For the sedimentation experiments, the tracking of an individual particle started when the particle had sedimented onto a cell in the focal plane of the microscope. For the holographic optical tweezers experiments, the tracking of a particle started when the particle was bound to the macrophage membrane and the trap was turned off. Figure 2 shows representative examples for the transport of phagosomes with a diameter of 1 µm, 2 µm and 3 µm. The resulting particle position time series were separated into segments with a length of 30 seconds^27^. Each segment was classified as a segment of active ballistic transport or as a segment of passive motion based on the travelled particle distance. The distance threshold for active ballistic transport was set to 400 nm, independent of whether the direction of motion was towards the nucleus or towards the periphery^27^. We found that a change of the threshold value of up to 75% had only negligible effects on the results of our phagosomal trajectory analysis (Figure S5).

For all trajectories, the following parameters were calculated: (i) the percentage of trajectories that show a strong centrifugal motion, *p*_centrifugal_; (ii) the percentage of active centripetal motion (towards the nucleus), *p*_nuc_, the percentage of active centrifugal motion (towards the cell periphery), *p*_per_ and the percentage of passive phases, *p*_pass_; (iii) the instantaneous velocity, *v*_inst_; (iv) the total active transport distance, *d*_tot_; (v) the total duration of active motion, *t*_tot_; (vi) the effective velocity, *v*_eff_; (vii) the persistence of motion, *p*; and (viii) the percentage of extended pause stages, *p*_ext-pause_.

The percentage of trajectories that show a strong centrifugal motion, *p*_centrifugal_ (i), was determined by calculating the share of trajectories in which the particles were displaced from the perinuclear region by more than 5 µm after the particle had already reached the nucleus. The displacement was measured by calculating the distance between the particle surface and the border of the nucleus. Figure 2A shows an example of a phagosomal trajectory that contains a strong centrifugal motion after the nucleus was reached. The percentages of active motion towards the nucleus, *p*_nuc_, and of active motion towards the periphery, *p*_per_ (ii), were determined by subdividing all active segments into segments of motion towards the nucleus and towards the cell periphery, depending on the direction of the motion. The remaining percentage of segments with passive phases, *p*_pass_, was calculated directly from these values (*p*_pass_ = 1 − *p*_nuc_ − *p*_per_). Figure 2C shows an example of a phagosomal trajectory with a high percentage of active motion towards the nucleus before the nucleus is reached. The instantaneous velocity, *v*_inst_, of every active segment (iii) was determined by fitting a line to the trajectory in the segment. The total active transport distance, *d*_tot_ (iv), was determined by adding up the positive and negative transport distances of all active segments of the trajectory. The total duration of active motion, *t*_tot_ (v), was determined by multiplying the number of active segments with the time duration of a segment (30 seconds). The effective velocity, *v*_eff_, of the active transport (vi) was calculated by taking the ratio of the total active transport distance and the total duration of active transport, *v*_eff_ = *d*_tot_/*t*_tot_. We defined a positive effective velocity (vi) or instantaneous velocity (iii) for a phagosome movement in the direction towards the nucleus and a negative velocity for a movement in the direction towards the cell periphery. The persistence of motion, *p* (vii), was determined as follows: First, for each active segment, the percentage of cases in which two consecutive particle displacements exhibit the same direction of motion was determined. Then, this percentage was averaged over all active segments of the trajectory to yield the persistence of motion value, *p*. The percentage of extended pause phases, p_ext-pause_ (viii), before the nucleus is reached was defined as follows: A region consisting of three segments was considered as an extended pause region when the total particle displacement in this region was less than three times the threshold for active motion. Figure 2B shows an example of an extended pause phase in the trajectory of a phagosome with a diameter of 2 µm.

To characterize the initial phagosome transport from the cell periphery to the perinuclear region, the parameters (ii) and (viii) were only calculated up to the first contact between the particle and the nucleus. For all other parameters, the analyzed trajectory depends on the transport characteristic. For phagosomes without strong centrifugal motion (i), the analyzed trajectory was identical to phagosome transport until the nucleus was reached for the first time. The end time point, t_end_, of the trajectory was set to the first contact between the phagosome and nucleus. Figure 2C shows an example for a trajectory without strong centrifugal motion. The end time point in this example was therefore t_end_ = 1084 s. For phagosomes with strong centrifugal motion (i), the end time point of the analyzed trajectory was set to 10 min after the time point when the threshold for strong centrifugal motion was exceeded for the first time, t_Th-cf-motion_. Figure 2A shows an example for a trajectory with strong centrifugal motion. The time point when the threshold for strong centrifugal motion was first exceeded was t_Th-cf-motion_ = 2085 s. The end time point was therefore t_end_ = t_Th-cf-motion_ + 10 min = 2685 s. For the parameters displayed in Fig. 6, the instantaneous velocity was calculated for phagosome transport from the cell periphery until the perinuclear region was reached.

#### Dynein motor distribution

For analyzing the dynein motor distribution around the phagosomes, the focus plane of a phagosome in the fluorescence image stack with labelled dyneins, as well as the plane above and below the focus plane, were projected along the axial direction. The projection yielded a two-dimensional image containing in each pixel the average intensity along the axial direction. We determined the particle position within the cell in the bright field image. At this position, we defined a circle with the diameter of the phagocytosed particle and determined the intensity along the circle outline. To decrease the noise due to fluorescence intensity fluctuations, the intensity distribution was averaged over a ring with an inner radius *r*_0_ and the outer radius *r*_0_ + Δ*r*, with Δ*r* = 0.64 μm and *r*_0_ = 0.5 μm,1.0 μm or 1.5 μm depending on the phagosome diameter. To compare the results of different particle sizes and for various phagosome positions the intensity distributions were calculated as a function of the polar angle φ. For each phagosome position, the angle φ = 0° corresponds to the direction towards the nucleus. The position of the nucleus was determined from the fluorescence image stack with a custom-written MATLAB program based on a threshold filter and the MATLAB *regionprops* algorithm. To compensate for variations in the fluorescence labelling efficiency, we normalized each angular intensity distribution to its maximal intensity. To quantify differences between the nucleus-facing side and the periphery-facing side of the phagosomes, we defined Δ*I*_dynein_ as the difference between the mean intensity for |φ| ≤ 45° and the mean intensity for |φ| ≥ 135°.

#### Microtubule distribution measurement

The microtubule distribution within the acquired cells was analyzed around existing phagosomes and around spheres with varying diameters. The analysis of microtubule distributions around phagosomes was identical to the analysis of the dynein motor distribution around phagosomes. For analyzing the microtubule distribution around spheres with varying diameters, image stacks of cells without phagosomes were projected along the axial direction (mean projection). Here, all image planes of the cell stack were used. Circles with diameters of 1 µm, 2 µm and 3 µm were defined at various randomly chosen positions inside the cells, and the intensity along the circle outlines was determined. The positions where the circles were located were randomly selected, with the boundary condition that the whole circle had to lie within the cytoplasm. The intensity distributions were calculated again as a function of the polar angle φ as described for the dynein labelling. To quantify differences between the nucleus-facing side and the periphery-facing side of phagosomes, we defined the value Δ*I*_MT-phagosomes_ analogously to the case of the dynein labeling. To quantify the differences between the nucleus-facing side and the periphery-facing side that phagosomes would be exposed to in the given microtubule distributions, we defined Δ*I*_MT-spheres_ as the difference between the mean intensity for |φ| ≤ 45° and the mean intensity for |φ| ≥ 135°.

### Data and materials availability

All data needed to evaluate the conclusions in the paper are present in the paper and/or the Supplementary Materials. Additional data related to this paper may be requested from the authors.

## Electronic supplementary material

Supplementary Information
