# Supplementary material for: Phagosomal transport depends strongly on phagosome size
Source: Sci Rep. 2017 Dec 6;7:17068. doi: 10.1038/s41598-017-17183-7 (PMC5719076; doi:10.1038/s41598-017-17183-7)
Supplement: Supplementary file 1 — Supplementary Information [file 41598_2017_17183_MOESM1_ESM.pdf]

Phagosomal transport depends strongly on phagosome size – Supplementary Materials

S. Keller*[1], K. Berghoff[1] and H. Kress[1]

[1]Department of Physics, University of Bayreuth, Universitaetsstrasse 30, D-95440 Bayreuth, Germany

*e-mail: steve.keller@uni-bayreuth.de

Supplemental Figures

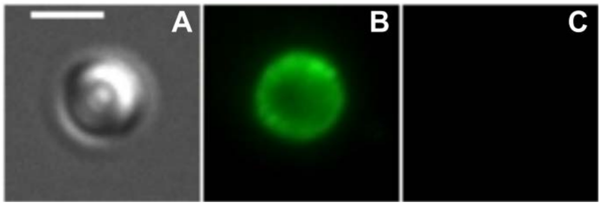

Figure S1. IgG-opsonization of the particles. The opsonization of the polystyrene microparticles was tested by using a fluorescently-labelled secondary antibody against the primary IgG antibody. Panel (A) shows a DIC image of a 3 µm-sized polystyrene particle opsonized with IgG and the fluorescent secondary antibody. Panel (B) shows the fluorescence signal of the secondary fluorescent antibody of the same particle. Panel (C) shows a particle without primary IgG antibody but with the secondary antibody imaged under the same conditions as the particle in panel (B). The scale bar is 3 µm.

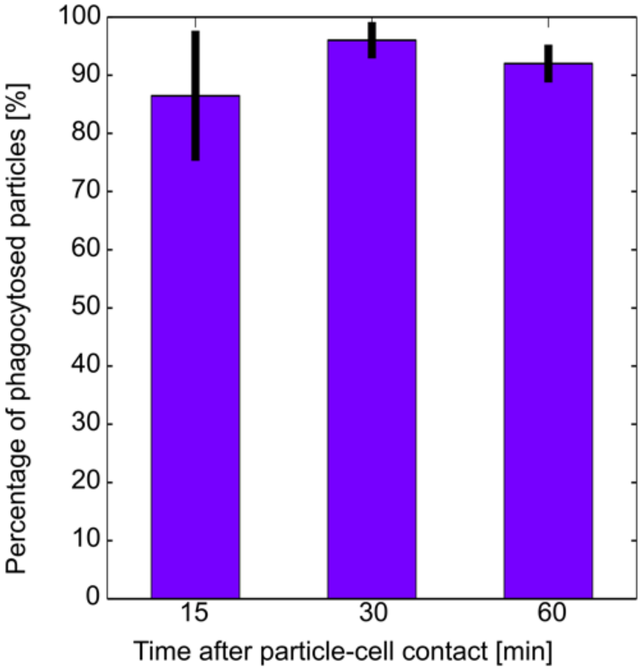

Figure S2. Probability of phagocytic uptake of IgG-opsonized particles. The figure shows the percentage of particles that were phagocytosed after they were in brought into contact with the cells for 15 min, 30 min, and 60 min, respectively. The number of analyzed particles was 539, 594 and 798 for the three different contact times of 15 min, 30 min, and 60 min. The particle diameter was 3 µm. The error bars show the standard deviation of different cell samples.

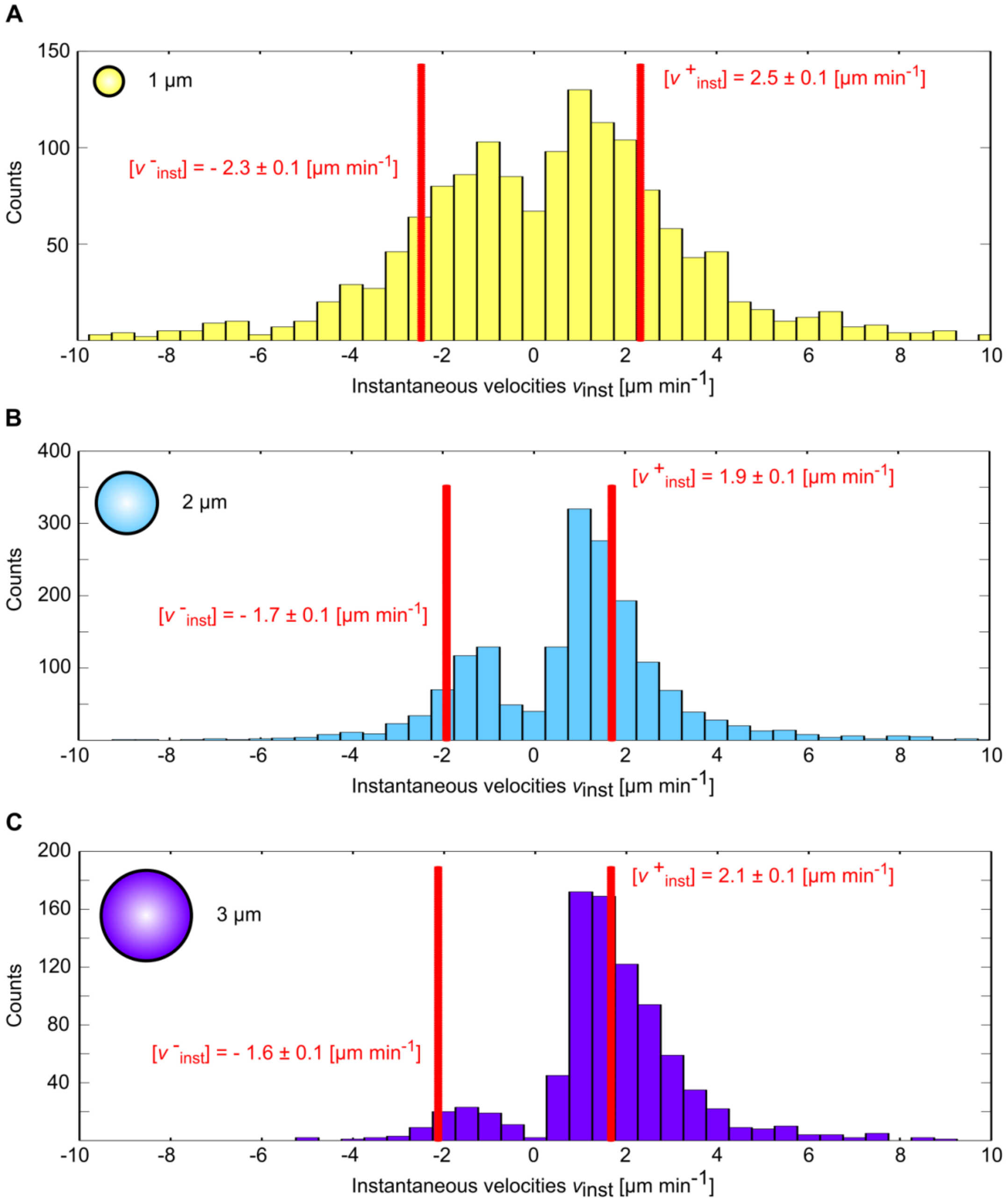

Figure S3. Distributions of the instantaneous phagosomal transport velocities of all active transport segments for phagosomes with a diameter of 1 µm (A), 2 µm (B), and 3 µm (C). Positive velocities are velocities for segments with an active transport towards the nucleus, and negative velocities represent segments with an active transport towards the cell periphery. The red lines and the displayed values shown in every panel represent the mean values (± std. error of mean) of the velocities in the two directions. For the small 1 µm-sized particles, the velocities in both directions are very similar to each other, whereas for the medium-sized 2 µm phagosomes and the large 3 µm particles, the transport towards the nucleus is significantly faster than the transport in the other direction. For 3 µm particles, the displayed mean velocities are significantly smaller than the instantaneous velocity depicted in Figure 3C. This difference arises because in Figure 3C, the mean instantaneous velocity of every single trajectory (every analyzed phagosome) is shown, whereas this figure shows the instantaneous velocities of every single active segment of all trajectories. The results represent 1450 active segments from 51 phagosome trajectories for 1 µm particles, 1742 active segments from 63 phagosome trajectories for 2 µm particles, and 869 active segments from 53 phagosome trajectories for 3 µm particles.

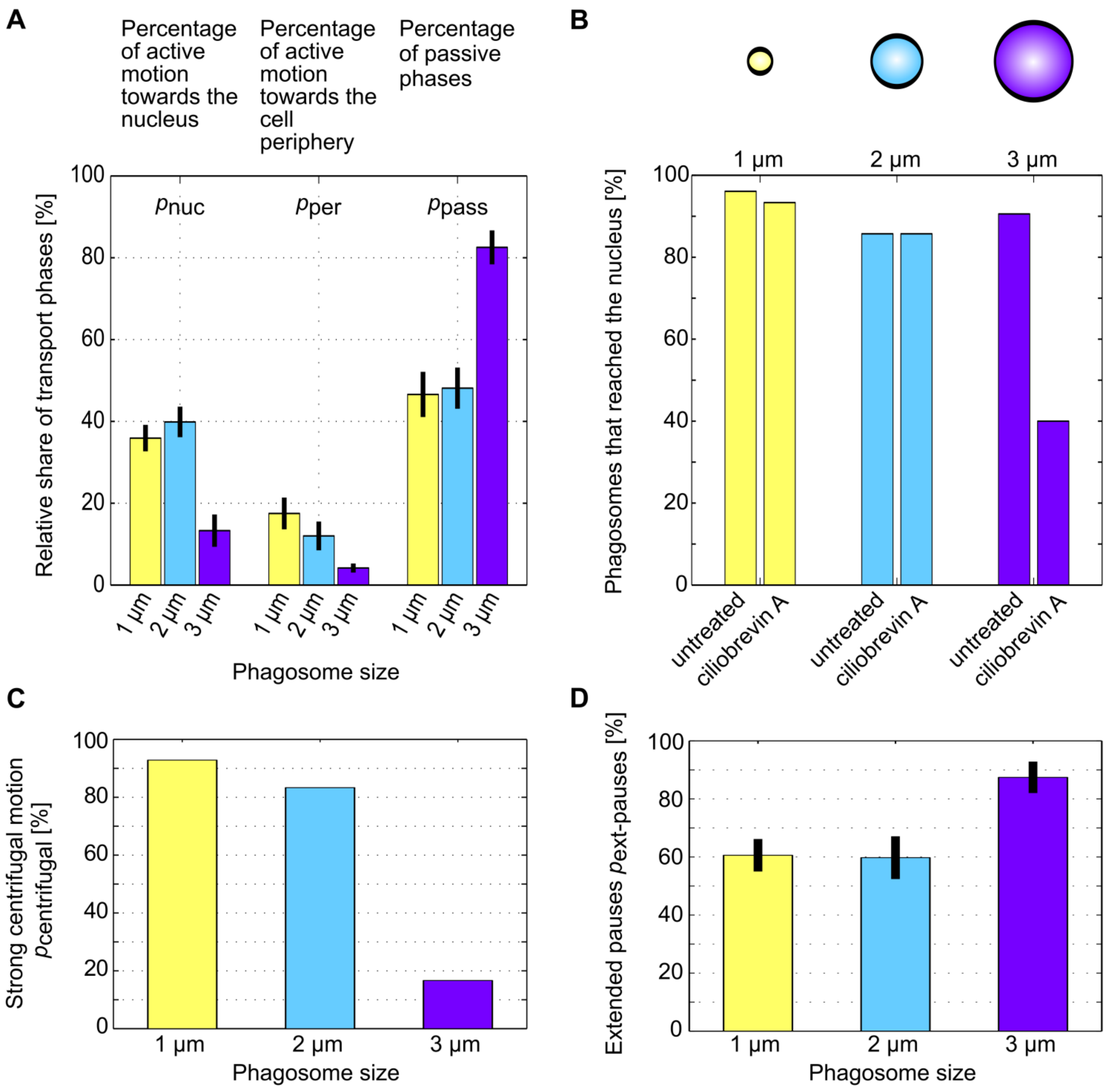

Figure S4. Influence of the dynein inhibiting drug ciliobrevin A on the size-dependent phagosomal transport. (A) Relative share of the different phases of the phagosomal transport for the three phagosome sizes in cells treated with 50 µM ciliobrevin A. (B) Percentage of phagosomes that reached the nucleus during the phagosome transport in untreated and ciliobrevin A-treated cells. (C) Percentage $p_{centrifugal}$ of phagosomes that showed a strong centrifugal motion after reaching the nucleus in ciliobrevin A-treated cells. (D) Percentage of extensive pause phases, $p_{ext\text{-}pause}$, in cells treated with ciliobrevin A. The error bars in all panels represent the std. error of the mean. We analyzed 51, 63, and 51 trajectories for 1 µm, 2 µm, and 3 µm phagosomes in untreated cells, and 15, 14, and 15 trajectories for 1 µm, 2 µm, and 3 µm phagosomes in ciliobrevin A-treated cells.

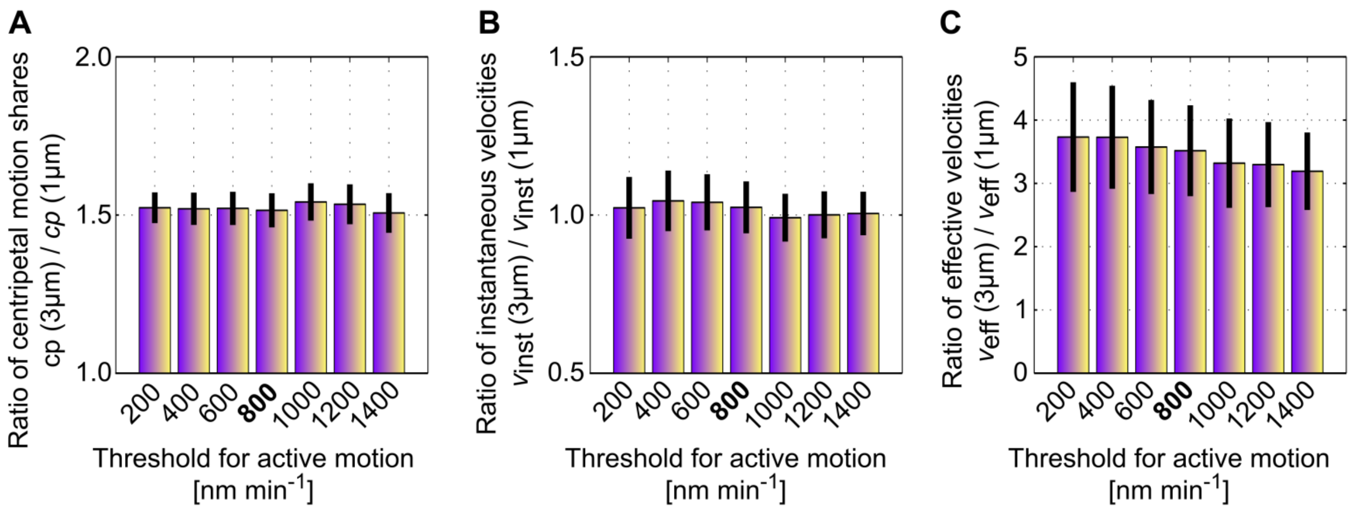

Figure S5. Correlation between various phagosomal transport characteristics and the threshold for active transport identification. Panel (A) shows the centripetal motion share of 3 µm particles, cp(3 µm), divided by the centripetal motion share of 1 µm particles, cp(1 µm). The centripetal motion share cp is defined as the percentage of segments with active motion towards the nucleus compared to all active transport segments. Panel (B) shows the ratio of the mean instantaneous velocities of 3 µm and 1 µm phagosomes. Panel (C) shows the ratio of the mean effective velocities of 3 µm and 1 µm phagosomes. Within the measurement uncertainties, all ratios are independent of the used threshold value, indicating that there is no correlation between the threshold for active transport identification and parameters for phagosomal transport characteristics. All ratios were calculated for seven thresholds between 200 and 1400 nm min^{-1}. For 1 µm phagosomes, 51 trajectories were analyzed, and for 3 µm phagosomes, 53 trajectories were analyzed. Error bars are the standard error of the mean.
